# Systematically Informed Literature Review: What is the Prevalence of Borderline Personality Disorder (BPD) in Adolescents, 13-17, using DSM-5 Criteria?

**DOI:** 10.1192/j.eurpsy.2024.472

**Published:** 2024-08-27

**Authors:** S. Abdullah

**Affiliations:** Child & Adolescent Psychiatry, CNTW NHS Foundation Trust, Newcastle upon Tyne, United Kingdom

## Abstract

**Introduction:**

In child & adolescent mental health settings, borderline personality disorder (BPD) is a dominant and substantial condition with high occurrence rates seen in community, crisis, and in-patient settings. Previously because of multiple concerns, BPD diagnosis in adolescents was considered questionable and was perceived to be invalid. However, in light of the evidence, recent guidelines and diagnostic manuals affirm the diagnosis in the under-18 population.

**Objectives:**

Given its existence in adolescents and that DSM-5 (from 2013) allows diagnosing BPD in adolescents, a study was conducted in 2019 to explore what current literature had to say about its prevalence.

**Methods:**

To answer this, a systematically informed literature review tried to look at the evidence. The hypothesis was that not many clinicians or researchers are aware of or using the opportunity to diagnose and thus manage BPD in adolescents, i.e., early in the course of this illness. Four databases were searched- PubMed, Embase, Medline, and Psycinfo- with the following inclusion & exclusion criteria:

1. Age: Adolescents (13-17).
2. BPD (disorder not traits or features).
3. Language – English, not just the abstract in English.
4. Time limit & diagnostic criteria (2013 onwards, DSM-5).
5. Full length articles not Abstracts alone.
6. No geographical limit.
7. Contacted academics personally for additional data.

Following search terms were used: Borderline Personality Disorder, BPD, EUPD, Emotionally Unstable Personality disorder, DSM V, DSM 5, Diagnostic and Statistical Manual of Mental Disorders 5, DSM-5, Prevalence, Rate.

**Results:**

All searches yielded 525 results. Other sources didn’t identify any other records to be included. Out of these 525 results, 74 were duplicates. The inclusion and exclusion criteria were applied on the remaining resources. Of the remaining records, 133 were in language other than English, and thus, were excluded. Remaining 318 articles were assessed for eligibility. Of these, 196 had used diagnostic criteria or rating scales based on previous diagnostic criteria, and thus were excluded. Furthermore, 41 articles had focused on a totally other clinical question than ours. 79 articles had the wrong age range as per our diagnostic criteria. Thus, the total number of articles which met inclusion and exclusion criteria was 02. The results showed higher rates of BPD in adolescents, especially in those exposed to online sexual solicitation (OSS) (355 vs 13%) and in females (80% of cases).

**Image:**

**Figure:**
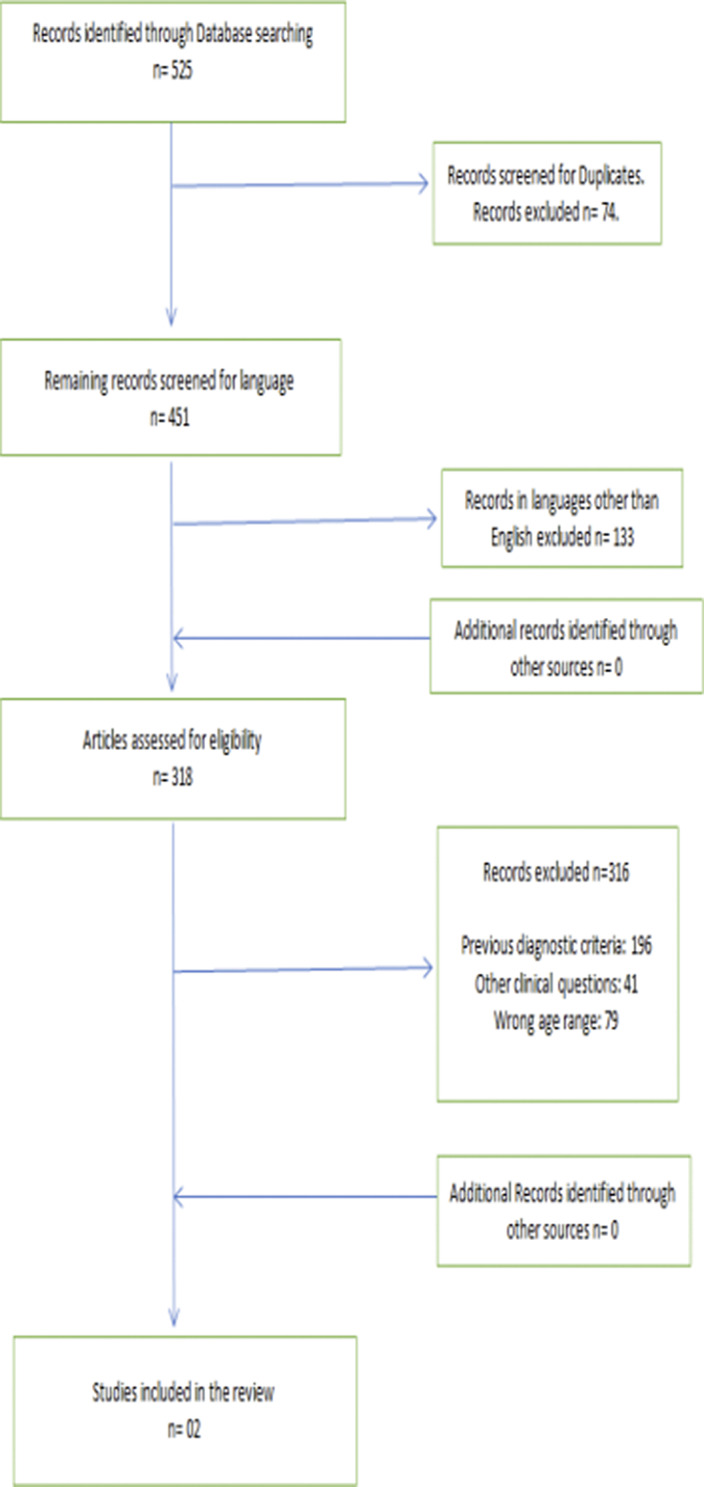

**Conclusions:**

Despite the research and diagnostic allowance, there still seems to be reluctance among clinicians to diagnose BPD in adolescents. We advise consideration of BPD in adolescents if clinical picture indicates and application of the relevant criteria so patients can get appropriate treatment and support that they need.

**Disclosure of Interest:**

None Declared

